# Two New Phenolic Glycosides from *Viscum articulatum*

**DOI:** 10.3390/molecules13102500

**Published:** 2008-10-15

**Authors:** Yang Li, Yan-Li Zhao, Ning Huang, Yong-Tang Zheng, Yong-Ping Yang, Xiao-Li Li

**Affiliations:** 1 Laboratory of Ethnobotany, Kunming Institute of Botany, Chinese Academy of Sciences, Kunming 650204, P. R. China; E-mail: liyang@mail.kib.ac.cn (Y. L.), zhaoyanli@mail.kib.ac.cn (Y-L. Z.); 2 Graduate University of Chinese Academy of Sciences, Beijing 100039, P. R. China; 3 Institute of Tibetan Plateau Research at Kunming, Kunming Institute of Botany, Chinese Academy of Sciences, Kunming 650204, Yunnan, P. R. China; 4 Key Laboratory of Animal Models and Human Disease Mechanisms, Kunming Institute of Zoology, Chinese Academy of Sciences, Kunming 650223; E-mail: huangning82@163.com (N. H.), zhengyt@mail.kiz.ac.cn (Y-T. Z.)

**Keywords:** *Viscum articulatum*, Phenolicglycoside, Flavanone

## Abstract

Two new phenolic glycosides, 1-*O*-benzyl-[5-*O*-benzoyl-*β*-d-apiofuranosyl (1→2)]-*β*-d-glucopyranoside (**1**), and 4′-hydroxy-7,3′-dimethoxyflavan-5-*O*-*β*-d-gluco-pyranoside (**2**), together with nine known flavanones **3 −11**, have been isolated from the dried whole plants of *Viscum articulatum*. Their structures were identified by extensive spectral analysis, especially 2D NMR techniques. Compound **9** showed weak anti-HIV-1 activity.

## Introduction

The genus *Viscum*, belonging to the Loranthaceae family, is a group of semi-parasitic shrubby type plants. Some species of this genus have shown to possess medicinal functions. The most well-known is mistletoe (*V*. *album* L.), which was frequently used as an alternative cancer treatment in Europe [1]. The plant of *V. articulatum* Burm. f., which has long been used as a folk herb, is distributed widely in the South and Southwest of China [2]. Previous investigations on *V. articulatum* showed that flavonoids, triterpenoids and organic acids were the major components of this plant [3,4,5,6,7], and some of them showed the inhibition effect on superoxide anion generation by human neutrophils in response to formyl-L-methionyl-L-leucyl-L-phenylalanin (fMLP)[6].

**Figure 1:**
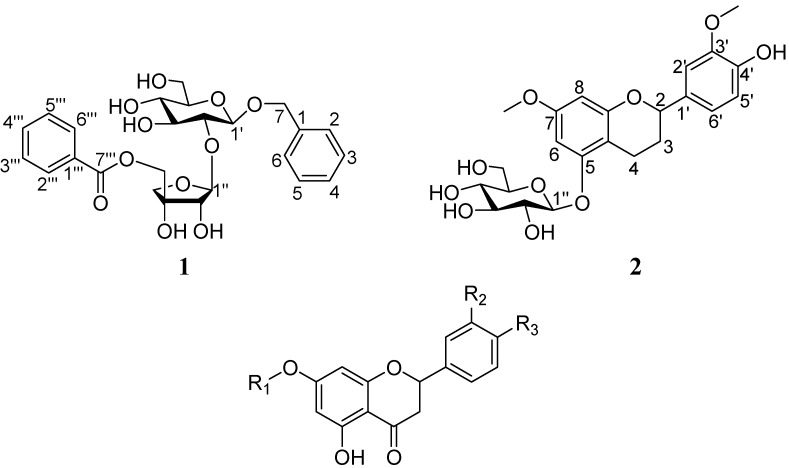
The structures of the isolated compounds 1 - 11 from *V. articulatum.*

**3 **R_1 _= -*β*-D-glucopyranosyl, R_2_=R_3_=H

**4 **R_1 _= -*β*-D-glucopyranosyl, R_2_=H, R_3_=OH

**5**R_1 _= -*β*-D-glucopyranosyl, R_2_=R_3_=OH

**6**R_1 _= -*β*-D-glucopyranosyl, R_2_=OMe, R_3_=OH

**7 **R_1 _= -[*β*-D-apiofuranosyl (1→2)]-*β*-D-glucopyranosyl, R_2_=R_3_=H,

**8 **R_1 _= -[cinnamoyl (1→5)-*β*-D-apiofuranosyl (1→2)]-*β*-D-glucopyranosyl, R_2_=R_3 _= H

**9** ****R_1 _= -*β*-D-glucopyranosyl, R_2_=OMe, R_3_= -*O*-*β*-D-apiofuranosyl

**10** R_1 _= -*β*-D-glucopyranosyl, R_2_=OMe, R_3_= -*O*-cinnamoyl (1→5)-*β*-D-apiofuranosyl

**11** R_1_ = -[*β*-D-apiofuranosyl (1→5)-*β*-D-apiofuranosyl (1→2)]-*β*-D-glucopyranosyl, R2=R3=H

Aiming at finding bioactive secondary metabolites, we chemically studied the aerial part of *V. articulatum*, which led to the isolation of two new glycosides: 1-*O*-benzyl-[5-*O*-benzoyl-*β*-d-apiofuranosyl(1→2)]-*β*-d-glucopyranoside (**1**) and 4′-hydroxy-7,3′-dimethoxyflavan-5-*O*-*β*-d-gluco-pyranoside (**2**), together with nine known flavonones: pinocembrin 7-*O*-*β*-d-glucopyranoside (**3**) [6], 5,4′-dihydroxyflavanone-7-*O*-*β*-d-lucopyranoside (**4**) [6], 5,3′,4′-trihydroxyflavanone-7-*O*-*β*-d-gluco-pyranoside (**5**) [6], homoeriodictyol-7-*O*-*β*-d-glucopyranoside (**6**) [3], pinocembrin-7-*O*-*β*-d-apio-furanosyl(1→2)-*β*-d-glucopyranoside (**7**) [4], pinocembrin-7-*O*-[cinnamoyl (1→5)-*β*-d-apiofuranosyl (1→2)]-*β*-d-glucopyranoside (**8**) [6], homoeriodictyol-7-*O*-*β*-d-glucopyranoside-4′-*O*-*β*-d-apio-furanoside (**9**) [3], homoeriodictyol-7-*O*-*β*-d-glucopyranoside-4′-*O*-*β*-d-(5′′′-cinnamoyl)apiofuranoside (**10**) [4], and pinocembrin-7-*O*-*β*-d-apiofuranosyl-(1→5)-*β*-D-apiofuranosyl-(1→2)-*β*-d-glucopyranoside (**11**) [4]. The structures of the new compounds were determined by means of spectral analysis and the known ones were identified by comparison of their NMR data with those reported in the literature. In addition, compounds **1, 3, 6 – 8** and **10 – 11** were tested for cytotoxicit**y** against the MDA-MB-435 and Hela cell lines and compounds **1 – 4, 6 – 11** were tested for anti-HIV activity. 

## Results and Discussion

**Figure 2:**
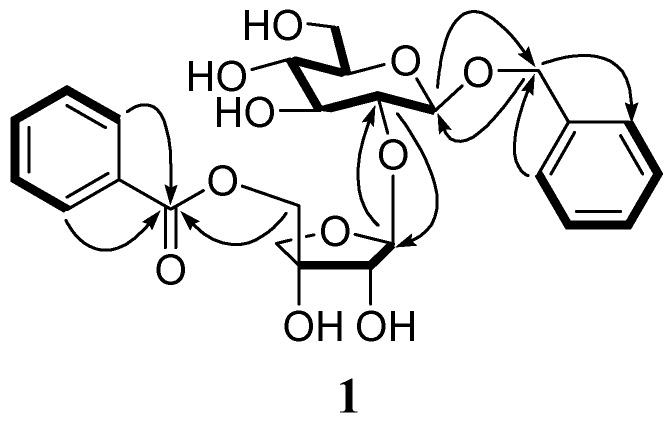
Key COSY (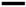) and HMBC (→) correlations of **1.**

Compound **1** was obtained as a pale yellow solid. Its molecular formula was determined as C_25_H_29_O_11_ on the basis of HR-ESI-MS data ([M − H]^−^, found 505.1711, calcd. 505.1709). However, initial observation of the ^13^C-NMR spectrum of **1** (Table 1) only showed 21 signals, assigned as one carbonyl carbon, four oxymethylene, seven oxymethines, six methines, one monooxygenated quaternary carbon, two quaternary carbons. The ^1^H-NMR spectrum of **1** (Table 1) displayed two sets of aromatic proton signals at *δ*_H_ 7.27 (d, *J* = 7.4 Hz, 2H), 7.20 (m, 2H) and 7.15 (m), as well as 7.94 (d, *J* = 7.6 Hz, 2H), 7.51 (m, 2H) and 7.65 (m). Their analysis in the HMQC spectrum revealed correlations of carbon signals at *δ*_C_ 127.7 (C-2 and C-6) with proton signals at *δ*_H_ 7.27, *δ*_C_ 128.1 (C-3 and C-5) with *δ*_H_ 7.20, *δ*_C_ 129.4 (C-2′′ and C-6′′) with *δ*_H_ 7.94, and *δ*_C_ 128.8 (C-3′′ and C-5′′) with *δ*_H_ 7.51. The chemical shifts and coupling constants of these signals, in combination with the observed ^1^H−^1^H COSY and HMBC correlations, suggested the presence of two monosubstituted aromatic rings (Figure 2). Therefore, 25 carbon signals were present in **1**, which supported the molecular formula obtained from HR-ESI-MS. Furthermore, the HMBC correlations from H-2 and H-6 to methylene [*δ*_C_ 69.7, C(7)] as well as from H-2′′′ and H-6′′′ to carbonyl carbon [*δ*_C_ 165.7, C(7′′′)] indicated the presence of a benzyl and a benzoyl moiety. Besides above signals, HMQC, ^1^H−^1^H COSY and HMBC correlations led us to assign signals at *δ*_H_ 3.10−5.34 and *δ*_C_ 61.1−108.5 as a glucose and an apiose. The HMBC correlations from H−C(1′′) (*δ*_H_ 5.34, brs) to C(2′) (*δ*_C_ 76.6) identified a apiofuranosyl (1→2) glucopyranosyl linkage. The benzyl and the benzoyl moiety were located at the C(1′) of the glucose and the C(5′′) of the apiose, respectively, which was confirmed by the HMBC correlations from H−C(1′) (*δ*(H) 4.31, d,*J* = 6.9) to methylene of the benzyl and from H−C(5′′) (H−C(5′′) (*δ*(H) 4.20 (d, *J* = 11.2 Hz), 4.29 (d, *J* = 11.2 Hz)) to C(7′′′) of the benzoyl.

The ^1^H- and ^13^C-NMR data of **1 **were similar to those of 2-*O*-*β*-d-apiosyl-d-glucose-1*β*,5′-dibenzoate [8,9]. The difference is that a methylene (*δ*_H_ 4.47 (d, *J* = 11.7 Hz), 4.77 (d, *J* = 11.7 Hz), *δ*_C_ 69.7) replaced one of the two carbonyl carbons in **1**, which was determined by HMBC correlation between H−C(1′) (*δ*_H_ 4.31 (d, *J* = 6.9 Hz) and methylene C(7). The *β*-anomeric configuration for the glucose was determined from a large ^3^*J*_H1__′, H2__′_ coupling constant value (6.9 Hz). The *β*-anomeric configuration for the apiose was indicated from the anomeric signals at *δ*_C_ 108.5 and a small ^3^*J*_H1__′′, H2__′′_ coupling constant [10]. Since only the d-configuration is known to exist in naturally occurring glucose and apiose [11], the sugars in **1** were tentatively assigned the d-configuration. Thus, the structure of **1 **was established as 1-*O*-benzyl [5-*O*-benzoyl-*β*-d-apiofuranosyl (1→2)]-*β*-d-glucopyranoside.

**Table 1:** ^1^H- and ^13^C-NMR Data of **1** (DMSO-d_6_).

Position	*δ*(C)	*δ*(H)	Position	*δ*(C)	*δ*(H)
C(1)	137.7 (s)	−	Api:		
H−C(2, 6)	127.7 (d)	7.27 (2H, d, *J* = 7.4)	H−C(1′′)	108.5 (d)	5.34 (brs)
H−C(3, 5)	128.1 (d)	7.20 (2H, m)	H−C(2′′)	76.3 (d)	3.82 (d, *J* = 5.7)
H−C(4)	127.4 (d)	7.15 (m)	C(3′′)	77.4 (s)	−
CH_2_(7)	69.7 (t)	4.47 (d,*J* = 11.7)	CH_2_(4′′)	73.9 (t)	3.63 (d,*J* = 9.4)
		4.77 (d,*J* = 11.7)			3.91 (d,*J* = 9.4)
Glc:			CH_2_(5′′)	67.8 (t)	4.20 (d, *J* = 11.2)
H−C(1′)	100.3 (d)	4.31 (d, *J* = 6.9)			4.29 (d, *J* = 11.2)
H−C(2′)	76.6 (d)	3.29−3.30	C(1′′′)	129.7 (s)	−
H−C(3′)	76.9 (d)	3.29−3.30	H−C(2′′′, 6′′′)	129.4 (d)	7.94 (2H, d, *J* = 7.6)
H−C(4′)	70.4 (d)	3.10−3.11	H−C(3′′′, 5′′′)	128.8 (d)	7.51 (2H, m)
H−C(5′)	77.2 (d)	3.10−3.11	H−C(4′′′)	133.4 (d)	7.65 (m)
CH_2_(6′)	61.1 (t)	3.45 (dd, 10.1, 5.1）	C(7′′′)	165.7 (s)	−
		3.69 (dd, 10.1, 5.1）			

^1^H- and ^13^C-NMR data were obtained at 500 and 100 MHz, respectively.

Compound **2** was obtained as pale yellow amorphous powder. Its molecular formula was determined as C_23_H_27_O_10_ on the basis of HR-ESI-MS data ([M − H]^−^, found 463.1615, calcd. 463.1604). The ^13^C-NMR spectrum (Table 2) showed the presence of twelve aromatic carbons (*δ*_C_ 93.6 −159.3), an oxymethine (*δ*_C_ 78.4), and two methylenes (*δ*_C_ 29.8, 20.1), indicating a flavane skeleton. In addition, the ^13^C-NMR spectrum displayed two methoxyl carbon signals at *δ*_C_ 55.8 and 56.3. Besides above data, a glucose moiety was established by the presence of signals at *δ*_H_ 3.41 − 4.90 and *δ*_C_ 71.4 − 102.0, in combination with analysis of HMQC and ^1^H−^1^H COSY spectrum. The connectivity of the glucose moiety to the flavane was designated by HMBC correlation from H−C(1′′) (*δ*(H) 4.90, d, *J* = 7.9 Hz) to C(5) (*δ*_C_ 158.5), which was further confirmed by NOESY correlation of anomeric proton H−C(1′′) with H−C(6).

**Figure 3:**
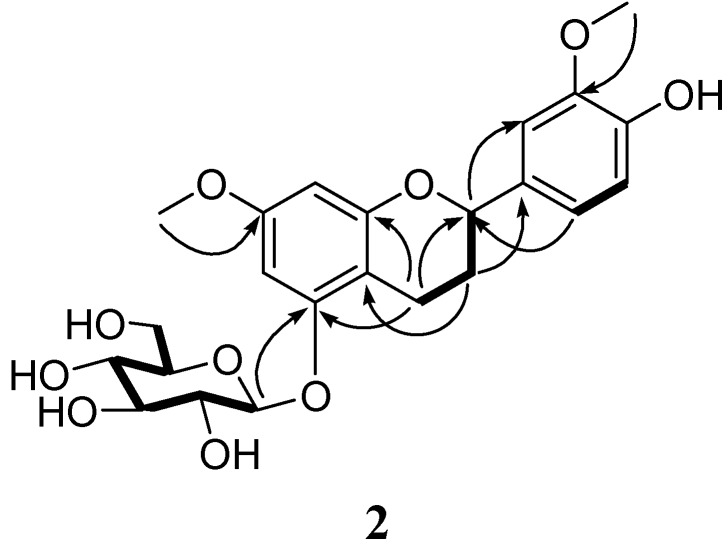
Key COSY (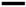) and HMBC (→) correlations of **2.**

The ^1^H−NMR spectrum showed that one of the aromatic rings was tetrasubstituted with two meta-coupled protons at *δ*_H_ 6.18 (s) and 6.28 (s) and the other trisubstituted, whose protons coupled in an ABX system at *δ*_H_ 6.81 (d, *J*=8.1 Hz), 6.87 (d, *J*=8.1 Hz) and 7.03 (s). The ^1^H- and ^13^C-NMR data of **2 **were similar to those of 7, 3′, 4′-dimethoxyflavan-5-*O*-*β*-D-glucopyranoside [12]. The difference was a hydroxyl replaced the methoxyl at C(4′) in **2**. In addition, two MeO-atoms located at C(7) and C(3′) were determined by HMBC correlations (Figure 3)of *δ*_H_ 3.78 (s, 3H) and 3.84 (s, 3H) with C(7) and C(3′) (*δ*_C_ 159.3 and 148.2), which was further confirmed by the NOESY correlations of *δ*_H_ 3.78 (s, 3H) with H−C(6) (6.18, s) and H−C(8) (6.28, s), and 3.84 (s, 3H) with H−C(2′) (7.03, s), respectively.

**Table 2:** ^1^H- and ^13^C-NMR Data of **2 **(acetone-d_6_).

Position	*δ*(C)	*δ*(H)	Position	*δ*(C)	*δ*(H)
H−C(2)	78.4 (d)	4.87 (m)	C(4′)	147.1 (s)	−
CH_2_(3)	29.8 (t)	1.95 (m)	H−C(5′)	115.5 (d)	6.81 (d, *J* = 8.1)
		2.13 (m)	H−C(6′)	119.8 (d)	6.87 (d, *J* = 8.1)
CH_2_(4)	20.1 (t)	2.59 (m)	MeO−(7)	55.8 (q)	3.78 (3H, s)
		2.65 (m)	MeO−(3′)	56.3 (q)	3.84 (3H, s)
C(5)	158.5 (s)	−	Glc:		
H−C(6)	97.9 (d)	6.18 (s)	H−C(1′′)	102.0 (d)	4.90 (d, *J* = 7.9)
C(7)	159.3 (s)	−	H−C(2′′)	74.7 (d)	3.41−3.44
H−C(8)	93.6 (d)	6.28 (s)	H−C(3′′)	77.7 (d)	3.48−3.51
C(9)	157.2 (s)	−	H−C(4′′)	71.4 (d)	3.41−3.44
C(10)	105.4 (s)	−	H−C(5′′)	78.0 (d)	3.48−3.51
C(1′)	134.2 (s)	−	CH_2_(6′′)	62.7 (t)	3.67 (dd, *J* = 10.2, 5.1）
H−C(2′)	110.8 (d)	7.03 (s)			3.86 (m)
C(3′)	148.2 (s)	−			

^1^H- and ^13^C-NMR data were obtained at 500 and 125 MHz, respectively.

Acid hydrolysis of **2** afforded d-glucose, which was identified by comparison of their *R_f_* with authentic sample. The *β*-anomeric configuration of the glucose was determined from a large ^3^*J*_H1__′′, H2__′′_ coupling constant value (7.9 Hz). Thus, the structure of **2 **was established as 4′-hydroxy-7,3′-dimethoxyflavan 5-*O*-*β*-d-glucopyranoside.

Compounds **1, 3, 6 – 8 **and **10 – 11** were tested for their anti-MDA-MB-435 and anti-Hela cell line activity by the MTT method, with cisplatin as positive control, but none of these compounds exhibited activity. Compounds **1 – 4, 6 – 11** were tested for cytotoxicity against C8166 cells (CC_50_), and anti-HIV-1 activity was evaluated by the inhibition assay for the cytopathic effects of HIV-1 (EC_50_), using AZT as a positive control. Compound **9** showed weak anti-HIV activity with CC_50_ > 200 μg/mL, EC_50_ = 18.09 μg/mL. Compound 9 exerted its weak protection of HIV**-**1_Ш__B_ inducted MT-4 host cells lytic effects with a TI > 11.06.

**Table 3:** Cytotoxicity and Anti-HIV-1 Activity of Compounds **1** - **4,** **6** - **11.**

Compound	Cytotoxicity, CC_50_ (ĉg/ml)	Anti-HIV-1 activity, EC_50_ (ĉg/ml)	TI
1	>200	112.05	>1.78
2	170.17	83.57	>2.04
3	>200	78.53	>2.55
4	>200	80.54	>2.48
6	>200	79.08	>2.53
7	>200	98.96	>2.02
8	>200	116.31	>1.72
9	>200	18.09	>11.06
10	>200	100.47	>1.99
11	>200	93.09	>2.15

### Experimental

#### General

Column chromatography (CC) was performed on silica gel (100 – 200 mesh; Qingdao Marine Chemical, Inc., P.R. China) and silica gel H (10 – 40 μm, Qingdao). Fractions were monitored by TLC, and spots were visualized by heating plates spraying with 10% H_2_SO_4_ in EtOH. UV Spectra: Shimadzu 210A double-beam spectrophotometer; *λ*_max_ log (*ε*) in nm. IR Spectra: Bio-Rad FTS-135 spectrophotometer, KBr discs; in *ν_max_* cm^–1^. Optical rotations: Horiba SEPA-300 spectro-polarimeter 1D- and 2D-NMR Spectra: Bruker AM-400 and DRX-500 instruments; chemical shifts *δ* in ppm rel. to residual solvent signals, *J* in Hz. ESIMS and HRESIMS: VG AutoSpec-3000 spectrometers.

#### Plant material

The whole plants of *V. articulatum*, semiparasites on the tree branches of *Lithocarpus variolosus* Chun, were collected in Lijiang county of Yunnan province in 2007 and verified by Prof. Yongping Yang and Prof. Zhekun Zhou. A voucher specimen (CJH 20070502-01) was deposited at Kunming Institute of Botany, Chinese Academy of Sciences, P. R. China.

#### Extraction and Isolation

The air-dried and powdered whole plants (1.5 kg) were extracted with 95% ethanol (3 × 10 L) for 24 h at room temperature and concentrated *in vacuo* to give a crude extract (85 g), which was suspended in H_2_O, and extracted successively with AcOEt. The AcOEt solution was evaporated, and the residue was directly subjected to column chromatography over MCI-gel CHP-20P eluting with 95% ethanol. The elute from 95% ethanol (67.5 g) was concentrated in *vacuo* and subjected to column chromatography over silica gel (200 − 300 mesh) eluting with petroleum ether and acetone step gradients to afford fractions A − E. Fraction C was repeatedly subjected to Sephadex LH − 20 and column chromatography over silica gel. Further purification with RP-18 yielded compound **1 **(6 mg), and repeated column chromatography over silica gel eluting with CHCl_3_−MeOH (9:1) yielded compound **2 **(11 mg)**, 3** (300 mg)**, 4** (5 mg)**, 5 **(2 mg)**, 6 **(2.1 g)**, 7 **(1.5 g)**, 8 **(30 mg)**, 9 **(14 mg)**, 10** (30mg)**, 11 **(38mg)**.**

*Compound* **1**: pale yellow solid; 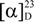 −50.0 (*c* 1.50, MeOH); UV (MeOH): 201 (0.58), 227(0.29), 274(0.04). IR (KBr): 3396, 2917, 1717, 1453, 1365, 1276, 1026, 825, 762, 718cm^−1^; ^1^H- and ^13^C-NMR: see Table 1. HR-ESI-MS (neg.) *m*/*z* 505.1711 (calcd for C_25_H_29_O_11_ [M − H]^−^, 505.1709).

*Compound* **2: **pale yellow amorphous powder; 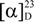 −31.4 (*c* 0.18, MeOH); UV (MeOH): 204(0.64), 280(0.05). IR (KBr): 3424, 2925, 1616, 1597, 1494, 1454, 1109, 821 cm^−1^; ^1^H- and ^13^C-NMR: see Table 2. HR-ESI-MS (neg.) m/z 463.1615 (calcd for C_23_H_27_O_10_ [M − H]^−^, 463.1604).

#### Acid Hydrolysis of **2**.

A solution of **2 **(8 mg) in 2 M HCl (3 mL) was heated in a water bath at 70 °C for 6 h. After cooling, the reaction mixture was neutralized with NaHCO_3_ and extracted with CHCl_3_. Through TLC comparison with an authentic sample using CHCl_3_-MeOH (8:2) as a developing system, D-glucose was detected in the water layer (*R_f_* = 0.16).

#### Cytotoxicity Assay

**Cytotoxi*c* activity was tested by MTT method with cisplatin as positive control [13] [14]. MDA-MB-435 and Hela cells were plated in the 96-well plate at a cell density of 5,000 cells per well and incubate at 37 °C for 24 h before treatment and continuously exposed to various concentrations of compounds. After 72 hours incubation, cell proliferation was analyzed by Cell Proliferation Kit I (MTT) according to the manufacturer’s instructions. The optical density of the wells was measured with a microplate reader at 570 nm. All assays were done in triplicate.

#### Anti-HIV-1 Assay

Cytotoxicity against C8166 (CC_50_) was assessed using the MTT method, and anti-HIV-1 activity was evaluated by the inhibition assay for the cytopathic effects of HIV-1 (EC_50_), with AZT as a positive control [15]. The assays included cytotoxicity in C8166 and MT-4 cells, inhibition of syncytium formation in HIV-1_Ш__B_-infected C8166 cells, and effect in protecting HIV-1_Ш__B_-infected MT-4 host cells from lytic effects in vitro.
